# Early Deterioration and Long-Term Prognosis of Patients With Intracerebral Hemorrhage Along With Hematoma Volume More Than 20 ml: Who Needs Surgery?

**DOI:** 10.3389/fneur.2021.789060

**Published:** 2022-01-05

**Authors:** Fuxin Lin, Qiu He, Youliang Tong, Mingpei Zhao, Gezhao Ye, Zhuyu Gao, Wei Huang, Lveming Cai, Fangyu Wang, Wenhua Fang, Yuanxiang Lin, Dengliang Wang, Linsun Dai, Dezhi Kang

**Affiliations:** ^1^Department of Neurosurgery, Neurosurgery Research Institute, The First Affiliated Hospital, Fujian Medical University, Fuzhou, China; ^2^Clinical Research and Translation Center, The First Affiliated Hospital, Fujian Medical University, Fuzhou, China; ^3^Fujian Clinical Research Center for Neurological Diseases, Fuzhou, China; ^4^Department of Neurosurgery, Wupin County Hospital, Wupin, China

**Keywords:** intracerebral hemorrhage, risk factors, prediction model, early deterioration, long-term prognosis

## Abstract

**Background and Purpose:** The treatment of patients with intracerebral hemorrhage along with moderate hematoma and without cerebral hernia is controversial. This study aimed to explore risk factors and establish prediction models for early deterioration and poor prognosis.

**Methods:** We screened patients from the prospective intracerebral hemorrhage (ICH) registration database (RIS-MIS-ICH, ClinicalTrials.gov Identifier: NCT03862729). The enrolled patients had no brain hernia at admission, with a hematoma volume of more than 20 ml. All patients were initially treated by conservative methods and followed up ≥ 1 year. A decline of Glasgow Coma Scale (GCS) more than 2 or conversion to surgery within 72 h after admission was defined as early deterioration. Modified Rankin Scale (mRS) ≥ 4 at 1 year after stroke was defined as poor prognosis. The independent risk factors of early deterioration and poor prognosis were determined by univariate and multivariate regression analysis. The prediction models were established based on the weight of the independent risk factors. The accuracy and value of models were tested by the receiver operating characteristic (ROC) curve.

**Results:** After screening 632 patients with ICH, a total of 123 legal patients were included. According to statistical analysis, admission GCS (OR, 1.43; 95% CI, 1.18–1.74; *P* < 0.001) and hematoma volume (OR, 0.9; 95% CI, 0.84–0.97; *P* = 0.003) were the independent risk factors for early deterioration. Hematoma location (OR, 0.027; 95% CI, 0.004–0.17; *P* < 0.001) and hematoma volume (OR, 1.09; 95% CI, 1.03–1.15; *P* < 0.001) were the independent risk factors for poor prognosis, and island sign had a trend toward significance (OR, 0.5; 95% CI, 0.16-1.57; *P* = 0.051). The admission GCS and hematoma volume score were combined for an early deterioration prediction model with a score from 2 to 5. ROC curve showed an area under the curve (AUC) was 0.778 and cut-off point was 3.5. Combining the score of hematoma volume, island sign, and hematoma location, a long-term prognosis prediction model was established with a score from 2 to 6. ROC curve showed AUC was 0.792 and cutoff point was 4.5.

**Conclusions:** The novel early deterioration and long-term prognosis prediction models are simple, objective, and accurate for patients with ICH along with a hematoma volume of more than 20 ml.

## Introduction

An intracerebral hemorrhage is a serious form of stroke with high mortality (30–40%) and disability (70–80%) (1). In recent years, many studies have proposed that the specific signs displayed on initial CT, such as black hole sign, island sign, swirl sign, and blood-fluid level in hematoma, are related to early hematoma expansion (HE) (2, 3). In addition, coagulation abnormalities, the interval from onset to initial CT, hematoma volume, intraventricular hemorrhage (IVH), are also associated with the expansion of the hematoma or perihematomal edema (PHE) (4). However, whether these signs can be used to predict the early deterioration or poor prognosis for patients with intracerebral hemorrhage (ICH) and as reliable indicators for early surgical intervention remains controversial.

The results of Surgical Trial in Intracerebral Hemorrhage (STICH) trials and minimally invasive surgery with thrombolysis in intracerebral haemorrhage evacuation (MISTIE) trials suggested that there were no significant differences between the prognosis of the early surgery group and the initial conservative treatment group in patients with ICH (5, 6). However, the subgroup analysis of the STICH II study showed that patients with the evacuation of hematoma within 21 h might have a better clinical prognosis (3). A meta-analysis of 8 studies with 2,186 patients demonstrated that surgery for hematoma removal within 8 h after a stroke can significantly improve the prognosis of patients with ICH (7). Theoretically, evacuating the hematoma before deterioration, eliminating the mass effect, and removing blood degradation products, could improve the prognosis of patients with early ICH. However, the treatment of patients with ICH along with hematoma volume more than 20ml and without cerebral hernia at admission is still controversial (8–11). Early screening and stratification may be helpful to identify patients with a high risk of deterioration and poor prognosis. Therefore, this study aimed to explore the risk factors and to establish a practical prediction model for early deterioration and poor prognosis.

## Methods

### Patients

This study recruited patients from our prospectively maintained patients-with-ICH database (RIS-MIS-ICH ClinicalTrials.gov Identifier: NCT03862729) between January 2015 and October 2019. The criteria for enrollment were as follows: (1) ICH diagnosed by emergent CT or computed tomographic angiography (CTA) within 24 h; (2) no cerebral hernia at admission and hematoma volume more than 20 ml; (3) no obstructive hydrocephalus caused by IVH; (4) patients with GCS score > 8 at admission; (5) initially treated by conservative approaches and no emergency surgical intervention was arranged. Ethical approval was obtained through the relevant ethics committee of the First Affiliated Hospital of Fujian Medical University (Ethical Approval Number: MRCTA, ECFAH of FMU [2018] 082-1). Additionally, this study also followed the relevant Chinese laws, regulations, and guidelines, as well as international laws and regulations.

### Treatment

The patients in this study were treated according to two guidelines, namely Chinese multidisciplinary expert consensus: Diagnosis and Treatment of Spontaneous Cerebral Hemorrhage (2015) and Guidelines for the Management of Spontaneous Intracerebral Hemorrhage: A Guideline for Healthcare Professionals From the American Heart Association/American Stroke Association (2015) (12). For patients with ICH along with hematoma volume more than 20 ml, with neurological dysfunction but without cerebral hernia, conservative treatment and minimally invasive surgery (MIS) were recommended as the first-line treatment. The consciousness and neurological functions of patients treated by conservative therapy were closely observed. If the GCS decreased 2 or more scores or the functional defect worsened, conversion to surgical treatment was recommended. CT scanning was performed in the conservative treatment group at the 6, 12, 24, 48, and 72 h after stroke.

### Data Collection and Endpoint

All the data retrieved from our prospective ICH patient registration database including: (1) personal information: gender, age, etc.; (2) history of present illness: time of onset, initial symptoms, pre-hospital management; (3) past medical history: hypertension, diabetes, anticoagulants, antiplatelet drugs, antihypertensive drugs, and other drugs; (4) physical examination: vital sign (blood pressure/respiration rates/temperature/heart rates), neurological examination (pupillary reflex, GCS, the strength of contra-lesional extremities, etc.); (5) laboratory assay: coagulation function, blood routine test, etc.; (6) radiological imaging: (a) initial CT: hematoma location, depth of hematoma, whether with IVH or not, hematoma volume, PHE, hematoma shape and density categorical scales score (13), black hole sign, island sign, swirl sign, blood-fluid level in a hematoma (2, 3). Additionally, hematoma shape and density categorical scales scores include 2 novel 5-point categorical scales, ranging from Category 1 (most regular shape and most homogeneous density) to Category 5 (most irregular shape and most heterogeneous density) (13); (b) repeat CT: expansion of hematoma and/or PHE, whether cerebral infarction occurred, whether hydrocephalus occurred; (7) post-hospitalization assessments: neurological examination daily, and mainly focused on GCS score and functional deficits; (8) follow-up data: all patients were followed up every 6 months after a stroke at the outpatient department or by the phone call. This study focused on mRS score, all-cause mortality, and follow-up events (re-hemorrhage, cerebral infarction, seizures, etc.) 1 year after stroke. Thus, HE was defined as a 33% increase in hematoma volume or an absolute increase of 6 ml in a repeat CT scan. Similarly, expansion of PHE was defined as an increase in the ratio of PHE volume to hematoma volume or an absolute increase of 5 ml in the repeat CT scan. The early deterioration was defined as a sudden decline of GCS more than 2 or conversion to surgical treatment within 72 h after admission. The poor prognosis was defined as mRS ≥ 4 at 1 year after stroke.

### Data Management and Quality Control

For data collection, data management, and quality control, refer to the registration information of the RIS-MIS-ICH study on the ClinicalTrials.gov website, which is as follows: https://www.clinicaltrials.gov/ct2/show/NCT03862729?term=fuxin$+$lin&draw=2&rank=2. The project-related data was collected through the electronic data registry system (Real Data Medical Research Inc.). (eRDDM syestem, Ningbo, Zhejiang Province, China) And the radiological image information and follow-up data were determined by two neuroradiologists with consensus.

### Statistical Analysis

Demographic and clinical characteristics were descriptively summarized as mean (*SD*) for continuous variables and as number (%) for categorical variables. The chi-square test and Fisher's exact test were used for categorical variables, while Student's *t*-test was used for continuous variables. Only the variables with a *P* < 0.1 on univariate analysis were enrolled in multivariate analysis. The independent risk factors of early deterioration and poor prognosis 1 year after stroke were determined by multivariate analysis, respectively. The corresponding scores were assigned based on the weights of each independent risk factor. Accordingly, a prediction model of early deterioration and prognosis 1 year after stroke was established. The receiver operating characteristic (ROC) curves were drawn and analyzed, to find the cut-off point and calculate the AUC. *P* < 0.05 was considered significant.

## Results

### Baseline Patient Characteristics and Outcomes

A total of 632 patients with ICH were screened (Supplementary Figure 1), of which only 376 patients were within 24 h from the onset to the initial CT. The results showed that 158 patients were without cerebral hernia at admission and with a hematoma volume of more than 20 ml. Among them, 35 patients chose surgical treatment as the primary treatment modality, while other 123 patients preferred the initial conservative treatment. However, only 86 (70%) patients from the initial conservative treatment group had complete follow-up data.

Among 123 patients who received initial conservative treatment, the average age was 58.1 ± 13 years old, including 93 men (75.6%) and 30 women (24.4%). The average admission GCS score was 11.7 ± 2.6, and the average time from onset to the initial CT scan was 11.5 ± 8.3 h. The initial CT showed that 89 patients (72.4%) had basal ganglia hemorrhage (deep), 38 patients (30.9%) had hematoma less than 1 cm from the cortex (superficial), and 46 patients (37.4%) had IVH. The average hematoma volume was 37.7 ± 14.6 ml, and the average PHE volume was 14.9 ± 14.4 ml. According to the previously published literature (13), the results of hematoma shape categorical scale scores were as follows: 21 (17.1%) scored 1, 44 (35.8%) scored 2, 35 (28.5%) scored 3, 17 (13.8%) scored 4, and 6 (4.9%) scored 5. The hematoma density categorical scale score of 1 to 4 were 56 (45.5%), 42 (34.1), 18 (14.6%), and 7 (5.7%), respectively. Based on morphologic features of hematoma on initial CT, the number of patients with black hole sign, island sign, swirl sign, the blood-fluid level was 36 (29.3%), 61 (49.6%), 20 (16.3%), and 7 (5.7%), respectively. During 72 h after admission, 62 (50.4%) were converted to surgical treatment, 67 (54.5%) suffered from early deterioration. The average GCS score at discharge and mRS score at discharge was 12.6 ± 3.3 and 3.3 ± 1.2, respectively. Among the 86 (70%) patients with complete follow-up data at 1 year, 27 (31.4%) had seizures, 3 (3.5%) had re-hemorrhage, and 1 (1.2%) had cerebral infarction. The 1-year-follow-up mRS score showed as follows: 15 (17.4%) scored 1, 22 (25.6%) scored 2, 9 (10.5%) scored 3, 27 (31.4%) scored 4, 6 (7%) scored 5, and 7 (8.1%) scored 6. Therefore, 40 patients (46.5%) had poor prognosis (mRS ≤ 4) (Table 1).

**Table 1:** Clinical characteristics of patients with intracerebral hemorrhage (ICH).

**Clinical characteristics**	**Number(proportion,%)**
Patients number	123
Age (year)	58.1 ± 13.0
**Gender**	
Male	93 (75.6)
Female	30(24.4)
**Hypertension**	
Yes	89 (72.4)
No	34(27.6)
**Diabetes**	
Yes	12 (9.8)
No	111(90.2)
**Oral anticoagulants**	
Yes	3 (2.4)
No	120 (97.6)
**Oral antiplatelet drugs**	
Yes	2(1.6)
No	121(98.4)
SBP (mmHg)	157.1 ± 25.9
DBP (mmHg)	90.3 ± 15.0
Admission GCS score	11.7 ± 2.6
INR	1.0 ± 0.1
APTT	29.6 ± 8.1
Time from onset to initial CT (h)	11.5 ± 8.3
**Hematoma Location**	
Basal ganglia	89 (72.4)
Lobar	34 (27.6)
**Hemisphere**	
Left	61(49.6)
Right	62(50.4)
**Depth of Hematoma**	
>1 cm	85(69.1)
≤ 1 cm	38(30.9)
**IVH**	
Yes	46 (37.4)
No	77 (62.6)
Hematoma volume (ml)	37.7 ± 14.6
PHE Volume (ml)	14.9 ± 14.4
Volume of hematoma+PHE (ml)	52.5 ± 22.9
**Hematoma shape categorical scale score**	
1	21 (17.1)
2	44 (35.8)
3	35 (28.5)
4	17 (13.8)
5	06 (4.9)
**Hematoma density categorical scale score**	
1	56 (45.5)
2	42 (34.1)
3	18 (14.6)
4	7 (5.7)
5	0 (0.0)
**Black hole sign**	
Yes	36 (29.3)
No	87 (70.7)
**Island sign**	
Yes	61 (49.6)
No	62 (50.4)
**Swirl sign**	
Yes	20 (16.3)
No	103 (83.7)
**Blood-fluid level in hematoma**	
Yes	7 (5.7)
No	116 (94.3)
**Converted to surgical intervention**	
Yes	62 (50.4)
No	61 (49.6)
**Pulmonary infection**	
Yes	66 (53.7)
No	57 (46.3)
**Cardiac events**	
Yes	6 (4.9)
No	117 (55.1)
**Seizures**	
Yes	2 (1.6)
No	121 (98.4)
GCS score at discharge	12.6 ± 3.3
mRS score at discharge	3.3 ± 1.2
**Disease deterioration during hospitalization**	
Yes	67 (54.5)
No	56 (45.5)
**Follow-up**	
Number of registered follow-up	86 (70.0)
Number of lost to follow -up	37 (30.0)
**Follow-up events**	
Seizures	27 (31.4)
Rehemorrhage	3 (3.5)
Cerebral infarction	1 (1.2)
**mRS score in 1 year after hemorrhage**	
1	15 (17.4)
2	22 (25.6)
3	9 (10.5)
4	27 (31.4)
5	6 (7.0)
6	7 (8.1)
**Prognosis in 1 year after hemorrhage**	
Good	46 (53.5)
Poor	40 (46.5)

### Risk Factors for Early Deterioration and Prediction Model

**Figure 1:**
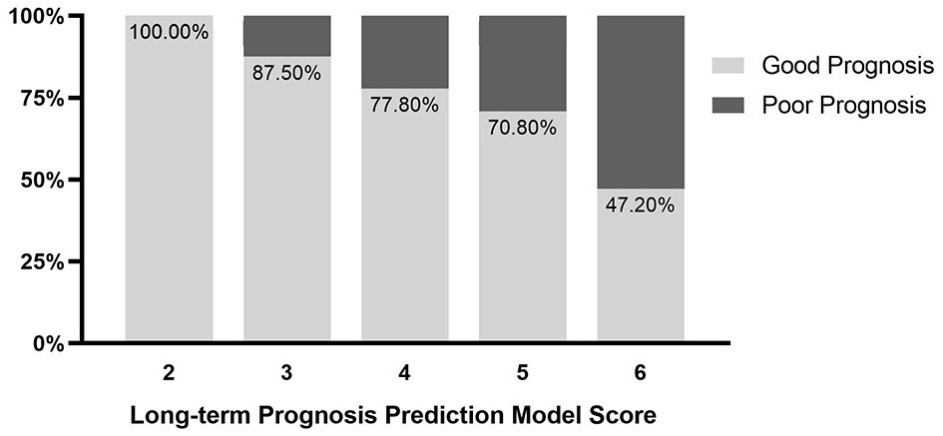
Probability distribution for early deterioration of patients with intracerebral hemorrhage (ICH) with different scores.

The univariate analysis indicated that admission GCS score (OR (odds ratio), 1.35; 95% CI,1.16-1.58; *P* < 0.001), time from onset to initial CT (OR, 1.04; 95% CI, 0.1–1.09; *P* = 0.061), hematoma volume (OR, 0.91; 95% CI, 0.88–0.95; *P* < 0.001), volume of hematoma and PHE (OR, 0.97; 95% CI, 0.95–0.98; *P* < 0.001), hematoma shape categorical scale score (OR, 0.66; 95% CI, 0.46–0.94; *P* = 0.021), black hole sign (OR, 2.05; 95% CI,0.91-4.6; *P* = 0.083), island sign (OR, 2.84; 95% CI, 1.36–5.92; *P* = 0.005) were the risk factors for early deterioration of patients with ICH. Then, these factors were included in the Logistic multiple regression model. The results showed that admission GCS score (OR, 1.43; 95% CI, 1.18–1.74; *P* < 0.001) and hematoma volume (OR, 0.9; 95% CI, 0.84–0.97; *P* = 0.003) were the independent risk factors (Table 2). Given the statistical analysis results and facilitating clinical practice, 13 < GCS ≤ 15 was assigned a score of 1, 10 < GCS ≤ 13 was assigned a score of 2, and 8 ≤ GCS ≤ 10 was assigned a score of 3. Additionally, hematoma volume ≤ 30 ml was assigned a score of 1, and volume of hematoma > 30 ml was assigned a score of 2. A scoring model for early deterioration was constructed to predict disease progression of patients with ICH at the early stage (Table 3). ROC curve analysis indicated that the prediction model is strongly associated with the early deterioration of patients with ICH, with an AUC of 0.778 and a cut-off point of 3.5 (Supplementary Figure 2). In brief, for the patients with ICH with a score of 2 (13.3%) or 3 (36.4%), the risk of deterioration during hospitalization was low, but for patients with a score of 4 (67.6%) or 5 (86.7%), the risk of deterioration during hospitalization was higher (Figure 1).

**Table 2:** Univariate logistic regression and multiple logistic regression of correlation between initial CT and an early deterioration.

**Variable**	**Univariate logistic regression**			**Multiple logistic regression**		
	**OR**	**95% CI**	***P* value**	**OR**	**95% CI**	***P* value**
Age	1.01	0.98–1.04	0.493			
Gender	1.27	0.56–2.89	0.572			
Hypertension	0.78	0.32–1.74	0.550			
Diabetes	2.74	0.71–10.67	0.146			
Oral anticoagulants	1.69	0.15–19.17	0.671			
Oral antiplatelet drugs	1.37	0.21–9.06	0.744			
SBP	0.99	0.98–1.01	0.857			
DBP	1.02	0.99 −1.04	0.201			
Admission GCS score	1.35	1.16–1.58	<0.001	1.43	1.18–1.74	<0.001
INR	2.10	0.04–17.24	0.717			
APTT	1.01	0.949–1.07	0.808			
Time from onset to initial CT (h)	1.04	0.10–1.09	0.061	1.03	0.97–1.09	0.418
Hematoma location	1.51	0.68–3.34	0.309			
Hemisphere	0.97	0.48–1.97	0.934			
Depth of hematoma	0.96	0.44–2.06	0.906			
IVH	1.14	0.55–2.38	0.724			
Hematoma volume	0.91	0.88–0.95	<0.001	0.90	0.84–0.97	0.003
PHE Volume	0.99	0.96–1.01	0.368			
Volume of hematoma and PHE	0.97	0.95–0.98	<0.001	1.00	0.97–1.04	0.943
Hematoma shape categorical scale score	0.66	0.46–0.94	0.021	0.90	0.54–1.50	0.681
Hematoma density categorical scale score	0.74	0.49–1.12	0.153			
Black hole sign	2.05	0.91–4.60	0.083	0.83	0.28–2.49	0.736
Island sign	2.84	1.36–5.92	0.005	1.53	0.52–4.50	0.436
Swirl sign	1.03	0.39–2.69	0.959			
Blood-fluid level in hematoma	2.18	0.41–11.68	0.364			

*SBP, Systolic blood pressure; DBP, Diastolic blood pressure; GCS, Glasgow Coma Scale; INR, International normalized ratio; APPT, Activated partial thromboplastin time; IVH, Intraventricular hemorrhage; PHE, Perihemotomal edema*.

**Table 3:** Early deterioration prediction model and prognosis prediction model.

**Early deterioration prediction model**		**Prognosis prediction model**	
Admission GCS score	Score	Hematoma volume	Score
13 < GCS ≤ 15	1	≤ 25	1
10 < GCS ≤ 13	2	>25	2
8 ≤ GCS ≤ 10	3	Island sign yes/no	1/0
Hematoma Volume	Score	Hematoma location	Score
≤ 30	1	Lobar	1
>30	2	Basal ganglia	3

*GCS, Glasgow Coma Scale*.

### Risk Factors for Poor Prognosis and Prediction Model

**Figure 2:**
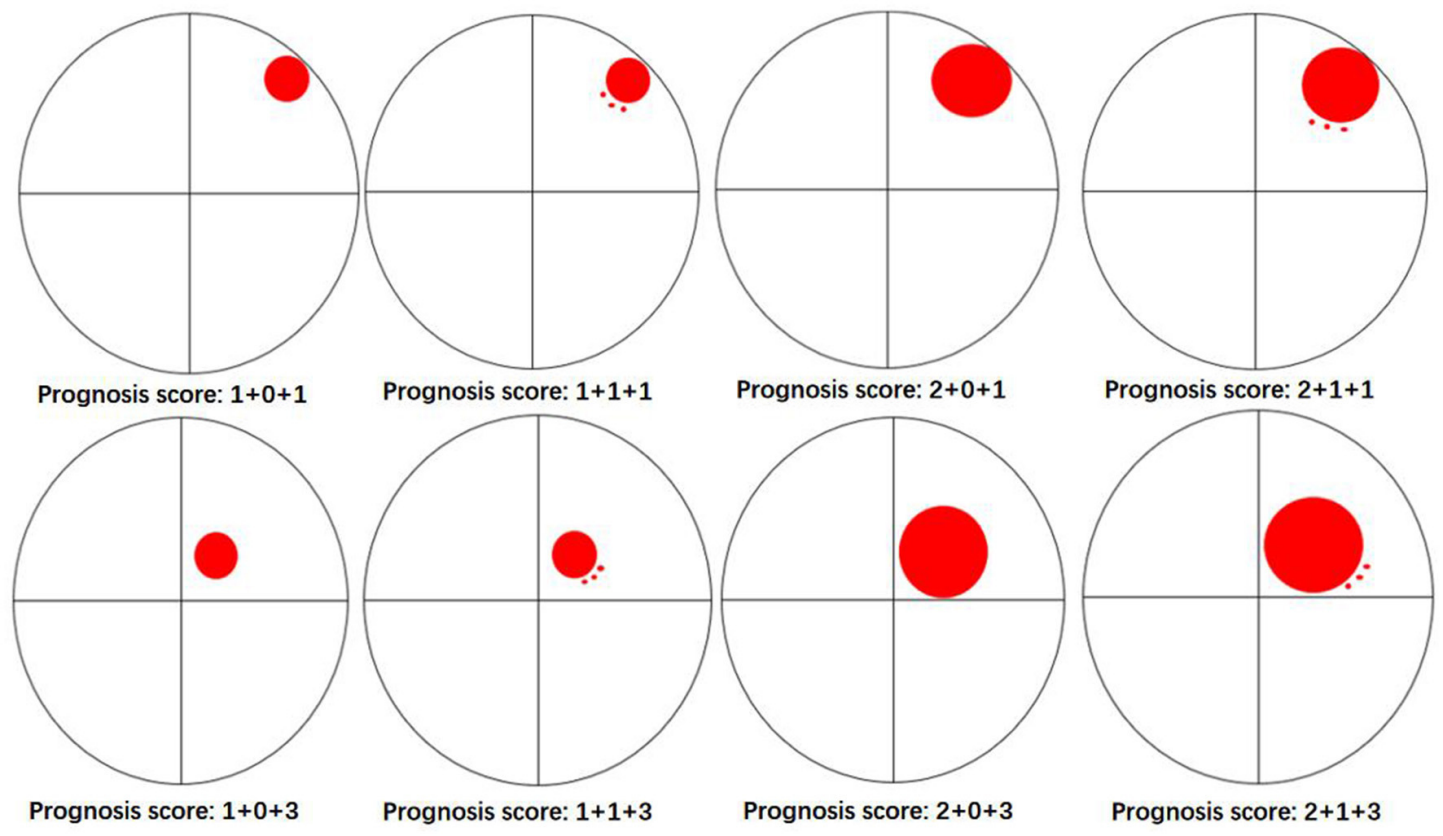
Probability distribution for poor prognosis of patients with ICH with different scores.

**Figure 3:**
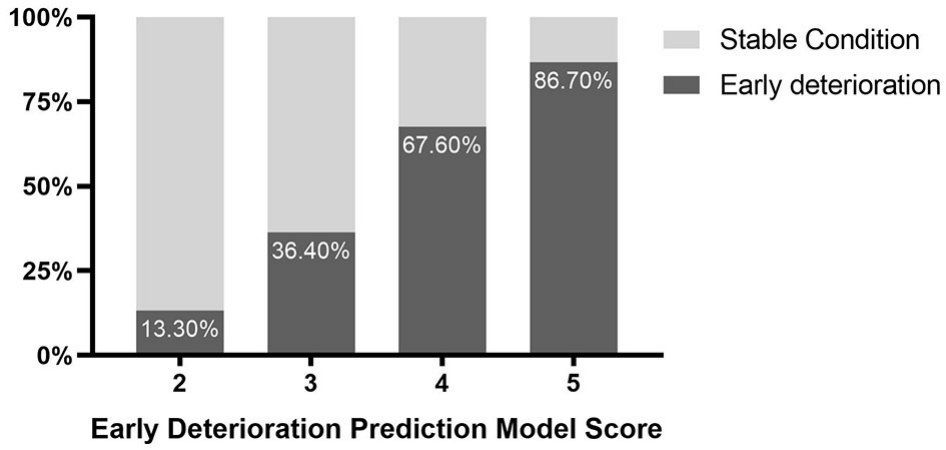
Schematic diagram of different scores in the prognosis prediction model.

The univariate logistic regression analysis indicated that with a history of hypertension (OR, 0.36; 95% CI, 0.13–1; *P* = 0.049), admission GCS score (OR, 0.8; 95% CI, 0.67–0.95; *P* = 0.012), hematoma location (OR, 0.07; 95% CI, 0.02–0.32; *P* = 0.001), depth of hematoma (OR, 0.28; 95% CI, 0.1–0.79; *P* = 0.016), hematoma volume (OR, 1.04; 95% CI, 1–1.08; *P* = 0.032), island sign (OR, 0.35; 95% CI, 0.15–0.85; *P* = 0.019) were the risk factors for the poor prognosis at 1 year after stroke. Then, these factors above were included in the multiple logistic regression model, and the results showed that hematoma location (OR, 0.027; 95% CI, 0.004–0.17; *P* < 0.001) and hematoma volume (OR, 1.09; 95% CI, 1.03-1.15; *P* <0.001) were independent risk factors. The island sign (OR, 0.5; 95% CI, 0.16–1.57; *P* = 0.051) had a trend to significant (Table 4). Based on the statistical analysis results and to facilitate clinical use, hematoma volume ≤ 25 ml was assigned a score of 1, and hematoma volume > 25 ml was assigned a score of 2. The lobar hematoma was assigned a score of 1, but basal ganglia hematoma was assigned a score of 3. Additionally, the appearance of the island sign on the initial CT scan was assigned a score of 1. Then, a scoring model for predicting the prognosis 1 year after stroke was constructed (Table 3). ROC curve analysis indicated that the prediction model was strongly associated with the prognosis of patients with ICH 1 year after stroke, with an AUC of.792 and a cut-off point of 4.5 (Supplementary Figure 3). In brief, for the patients with a score of 2 to 4, the risk of poor prognosis in 1 year after stroke was low, but for the patients with a score of 5 (29.2%) or 6 (52.8%), the risk of poor prognosis in 1 year after stroke was higher (Figure 2). To facilitate the clinical evaluation, the various types of prognostic scores were summarized in Figure 3.

**Table 4:** Univariate logistic regression and multiple logistic regression of correlation between initial CT and prognosis in 1 year after stroke.

**Variable**	**Univariate logistic regression**			**multiple logistic regression**		
	**OR**	**95% CI**	***P* value**	**OR**	**95% CI**	***P* value**
Age	1.03	0.99–1.07	0.112			
Gender	0.76	0.29–1.97	0.576			
Hypertension	0.36	0.13–1.00	0.049	0.19	0.04–0.86	0.235
Diabetes	0.54	0.14–2.07	0.368			
Oral anticoagulants	0.88	0.05–14.32	0.920			
Oral antiplatelet drugs	0.86	0.05–14.32	0.920			
SBP	1.01	0.99–1.02	0.374			
DBP	1.01	0.98 −1.04	0.435			
Admission GCS score	0.80	0.67–0.95	0.012	0.995	0.76–1.20	0.690
INR	0.09	0.00–11.04	0.328			
APTT	0.97	0.90–1.05	0.433			
Time from onset to Initial CT (h)	0.97	0.93–1.03	0.316			
Hematoma location	0.07	0.02–0.32	0.001	0.027	0.004–0.17	<0.001
Hemisphere	1.90	0.81–4.49	0.143			
Depth of hematoma	0.28	0.10–0.79	0.016			
IVH	0.54	0.22–1.28	0.161			
Hematoma Volume	1.04	1.00–1.08	0.032	1.09	1.03–1.15	<0.001
PHE Volume	1.00	0.97–1.03	0.978			
Volume of hematoma and PHE	1.01	0.99–1.03	0.197			
Hematoma shape categorical scale score	1.31	0.87–1.98	0.197			
Hematoma density categorical scale score	1.27	0.78–2.08	0.342			
Black hole sign	1.15	0.45–2.94	0.765			
Island sign	0.35	0.15 −0.85	0.019	0.50	0.16–1.57	0.051
Swirl sign	0.60	0.19–1.91	0.386			
Blood–fluid level in hematoma	1.33	0.21–8.36	0.764			

*SBP, Systolic blood pressure; DBP, Diastolic blood pressure; GCS, Glasgow Coma Scale; INR, International normalized ratio; APPT, Activated partial thromboplastin time; IVH, Intraventricular hemorrhage; PHE, Perihemotomal edema*.

## Discussion

The STICH trails are the milestones in the field of surgical invention study for ICH. STICH-I study did not find that early surgical treatment (within 72 h from onset) can benefit patients with supratentorial ICH but only suggested that patients with superficial hematoma (within 1 cm from the brain surface) may benefit from surgery (8). However, the STICH-II study focused on cerebral lobe hemorrhage revealed that early surgical treatment of patients within 12 h from onset did not reduce the death and disability rate of patients with lobar ICH. Therefore, the 2015 AHA/ASA guideline for the treatment of spontaneous ICH is recommended as follows: for most patients with supratentorial ICH, the usefulness of surgery is not well established; a policy of early hematoma evacuation is not clearly beneficial compared with hematoma evacuation when patients deteriorate. In recent years, a series of studies involved in MIS treatment for ICH had been conducted (8, 9). The MISTIE-II study verified the safety of MIS combined with rt-PA perfusion in the treatment of supratentorial patients with ICH with hematoma greater than 20 ml and within 72 h from a stroke. However, the results of the MISTIE-III study showed that for moderate to large intracranial hematomas, MIS combined with rt-PA perfusion did not improve the overall functional prognosis at 1 year after stroke (9). But, in our opinion, MIS plus rt-PA perfusion treatment seems not to be superior to conventional treatment modalities might be due to the failure of accurate stratification of enrolled patients. Accurate stratification of patients with ICH to identify the patients with a high risk of early deterioration and destined poor prognosis is important to invasive surgery decisions. Applying appropriate minimally invasive surgical methods to suitable subgroups of patients to interrupt the vicious circle after cerebral hemorrhage may be able to improve the functional prognosis for this subgroup of patients. Given that a hematoma volume < 20 mL manifested in little mass effect (14), the patients with ICH along with hematoma volume more than 20 ml without cerebral hernia were enrolled in this study.

Previous studies suggested that HE is associated with worse outcomes (15–17). Risk factors for the expansion of hematoma have become a major topic of hemorrhagic stroke research. In 2007, Wada R et al. proposed the correlation between early CTA imaging punctate enhancement (spot sign) and expansion of hematoma (18). From 2015 to 2017, blend sign, black hole sign, and island sign-on non-contrast CT were successively identified as predictors of the expansion of hematoma (19–21). In recent years, reduced perihematomal cerebral blood volume (CVB) by computed tomography perfusion (CTP) has been confirmed to be associated with HE (22). Compared with the spot sign on CTA, the specific radiological signs on non-contrast CT are more convenient in clinical. Some researchers hypothesized that the specific radiological signs may be associated with prognosis and could be used as evidence for early surgical intervention. In 2016, Gregoire B et al. suggested that low density of hematoma on emergency CT was related to the prognosis of patients with ICH (23). In 2018, Peter B. S et al. confirmed black hole sign, blend sign, island sign, hypodensities, and heterogeneous densities were reliable predictors of poor outcomes in patients with ICH (24). These findings are consistent with our results. We determined that the island sign was associated with the poor prognosis at 1 year after stroke and was used as an independent risk factor in the prognosis prediction model. However, the CT signs were directly related to the expansion of hematoma, but not directly related to the deterioration of the patients. This indirect relationship weakened the correlation between the CT sign and the patient's deterioration. Therefore, as the results of this study, the directly related factors such as admission GCS score and hematoma volume were more weighted than CT signs in the early deterioration prediction model.

According to the novel prediction models proposed in this study, patients with ICH with a score of 4 or 5 in the early deterioration prediction model will have a higher risk of early deterioration during hospitalization. Similarly, the patients with ICH with a score of 5 or 6 in the prognosis prediction model will suffer from a higher risk of poor prognosis at 1 year after stroke. In 2019, after integrative analysis of STICH trials and STITCH study patients, Gregson BA et al. found patients with a GCS score from 10 to 13 or a large ICH are likely to benefit from surgery (25). By comparing the patients from the ERICH study (test group) and ATACH-II study (validation group), Audrey C Leasure et al. concluded that 8 ml in thalamic and 18 ml in basal ganglia ICH as an optimal cut-off point for predicting the poor prognosis (26). These researches also supported our study results partly. GCS score < 13, hematoma volume > 25 ml, and basal ganglia hematoma were all independent risk factors and accounted for larger weights in our prediction models. Therefore, early MIS may be beneficial for patients with ICH with disorders of consciousness (GCS score ≤ 13), hematoma volume more than 25 ml, and elevated risk of hematoma expansion (island sign).

## Conclusions

The early deterioration and prognosis prediction model of the patients with ICH with hematoma volume more than 20 ml has the advantages of simplicity, objectivity, and accuracy, which may be helpful for clinicians to select treatment methods and prognosis consultation. Patients with an early deterioration score of 4 or 5 may have a higher risk of deterioration during hospitalization. Patients with a prognosis score of 5 or 6 may have a higher risk of poorer prognosis at 1 year after stroke. The prediction models will be validated by prospective multicenter large-sample-size data at the next step by the RIS-MIS-ICH study.

## Data Availability Statement

The raw data supporting the conclusions of this article will be made available by the authors, without undue reservation.

## Ethics Statement

The studies involving human participants were reviewed and approved by Ethics Committee of the First Affiliated Hospital of Fujian Medical University. The patients/participants provided their written informed consent to participate in this study.

## Author Contributions

DK has obtained research funding and is the principal investigator of the study. LD, FL, QH, YT, DK, and WF have developed this study, including ensuring ethical principles, designing study methodology, and drafting and revising the manuscript. MZ, GY, ZG, WH, and LC have participated in this study for data collection and follow-up. FW, DW, and YL have provided helpful feedback for all aspects of the work and participated in the final design of the study.

## Funding

Project on research and application of effective intervention techniques for a high-risk population of stroke from the National Health and Family Planning Commission in China (GN-2018R002); National Cerebrovascular and Nervous System Difficult Diseases Diagnosis and Treatment Capacity Improvement Project; Fujian Province High-level Neuromedical Center Construction Fund (Grant Number: HLNCC-FJFY-003).

## Conflict of Interest

The authors declare that the research was conducted in the absence of any commercial or financial relationships that could be construed as a potential conflict of interest.

## Publisher's Note

All claims expressed in this article are solely those of the authors and do not necessarily represent those of their affiliated organizations, or those of the publisher, the editors and the reviewers. Any product that may be evaluated in this article, or claim that may be made by its manufacturer, is not guaranteed or endorsed by the publisher.

## Supplementary Material

Supplemental Figure 1 Flow chart of patient selection. ICH, intracerebral hemorrhage; CT, Computed tomography.

Supplemental Figure 2 ROC curve analysis between admission GCS score and early deterioration, AUC was.703,cutoff point was 9.5 (green line). ROC curve analysis between hematoma volume of initial CT and early deterioration, AUC was.765, the cutoff point was 31.6ml (blue line). ROC curve analysis between hematoma volume of the early deterioration prediction model, AUC was.778, the cutoff point was 3.5 (red line).

Supplemental Figure 3 ROC curve analysis between hematoma volume and poor prognosis in 1 year after stroke, AUC was.627,cutoff point was 24.8 ml (blue line). ROC curve analysis of prognosis prediction model, AUC was.792, the cutoff point was 4.5 (green line).
