# Supplementary figures and images for: Early Deterioration and Long-Term Prognosis of Patients With Intracerebral Hemorrhage Along With Hematoma Volume More Than 20 ml: Who Needs Surgery?
Source: Front Neurol. 2022 Jan 5;12:789060. doi: 10.3389/fneur.2021.789060 (PMC8766747; doi:10.3389/fneur.2021.789060)

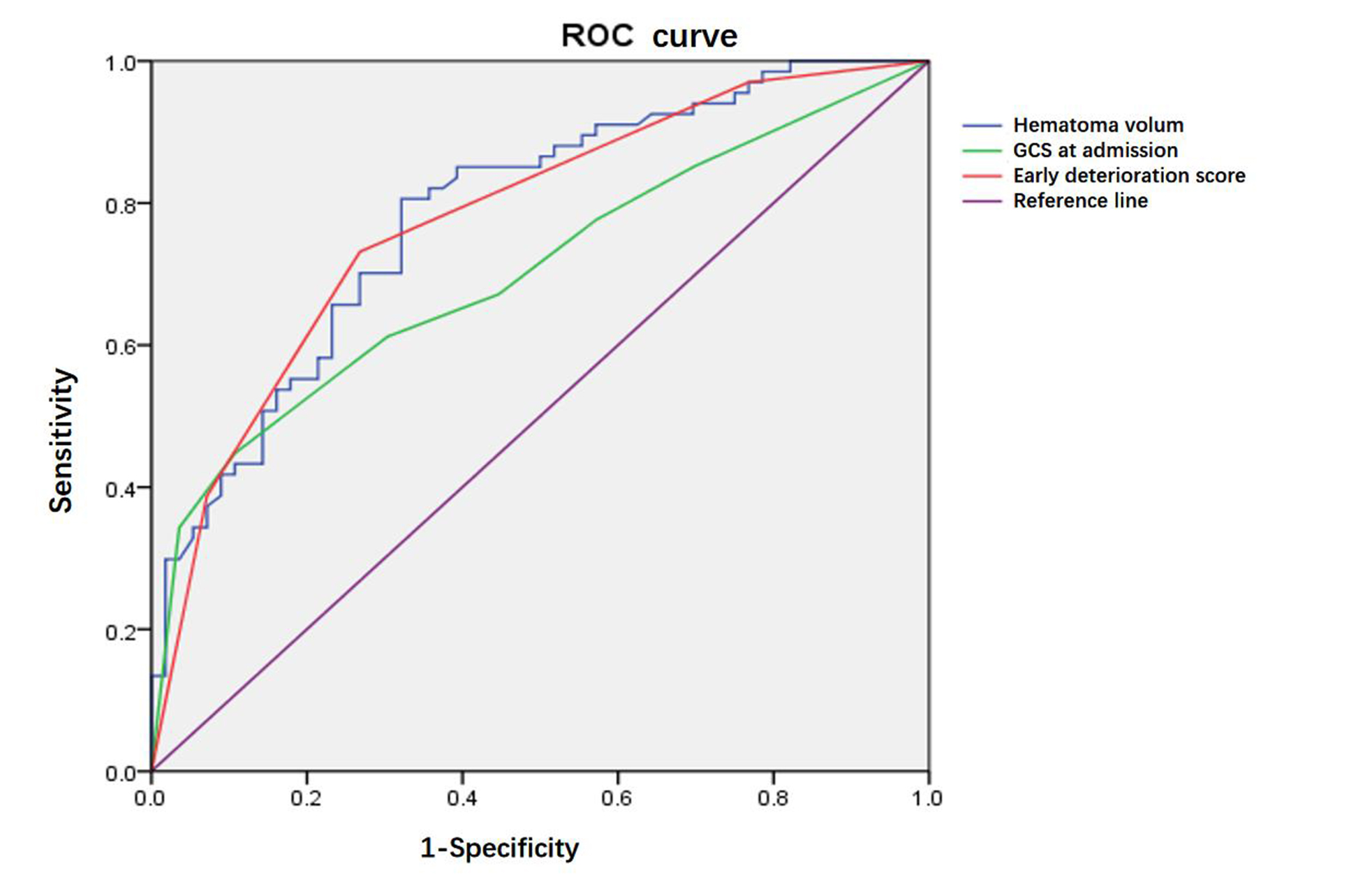

Supplement: Supplemental Figure 1 — Flow chart of patient selection. ICH, intracerebral hemorrhage; CT, Computed tomography. [file Image_1.JPG]

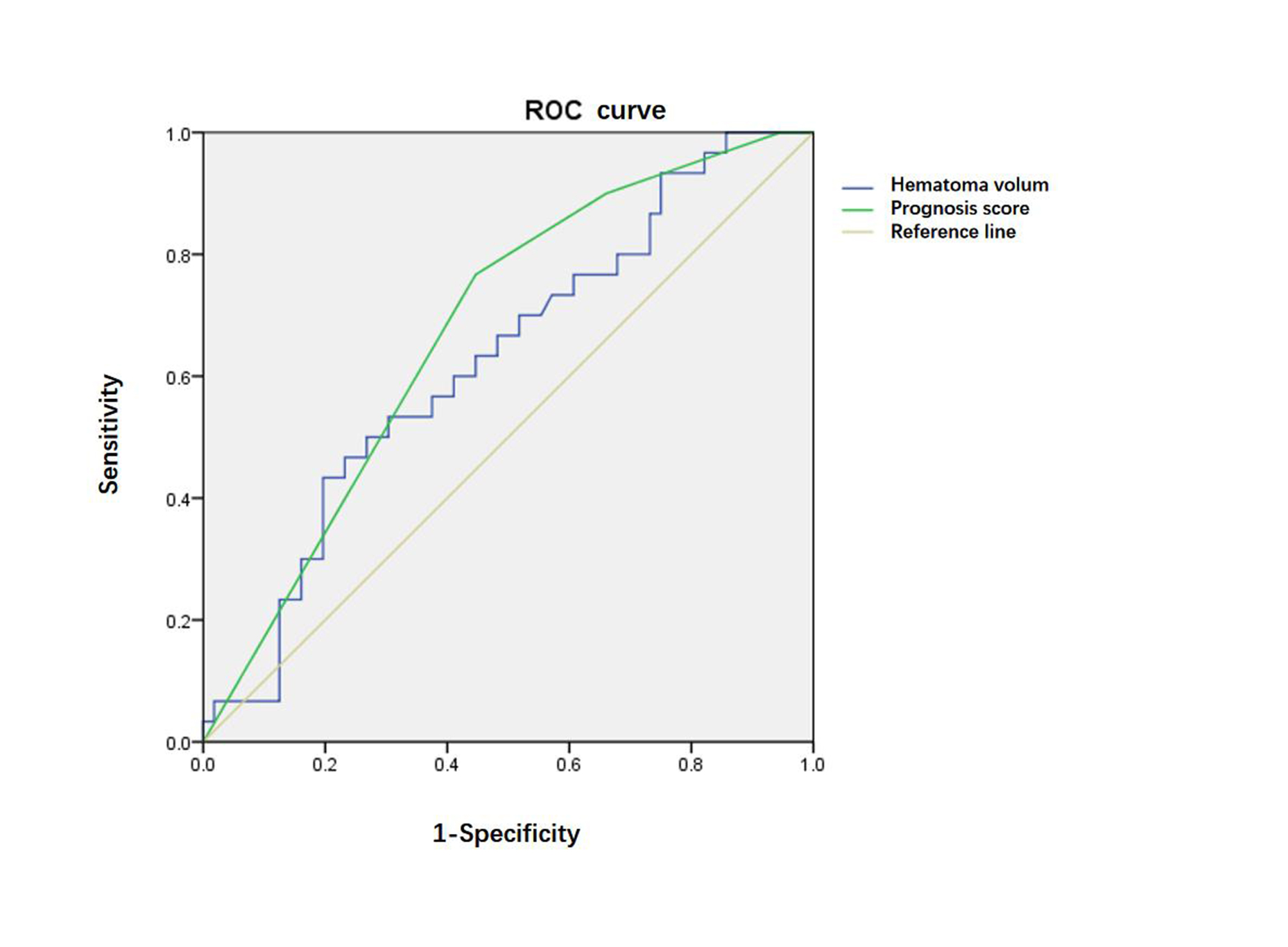

Supplement: Supplemental Figure 2 — ROC curve analysis between admission GCS score and early deterioration, AUC was.703,cutoff point was 9.5 (green line). ROC curve analysis between hematoma volume of initial CT and early deterioration, AUC was.765, the cutoff point was 31.6ml (blue line). ROC curve analysis between hematoma volume of the early deterioration prediction model, AUC was.778, the cutoff point was 3.5 (red line). [file Image_2.JPG]

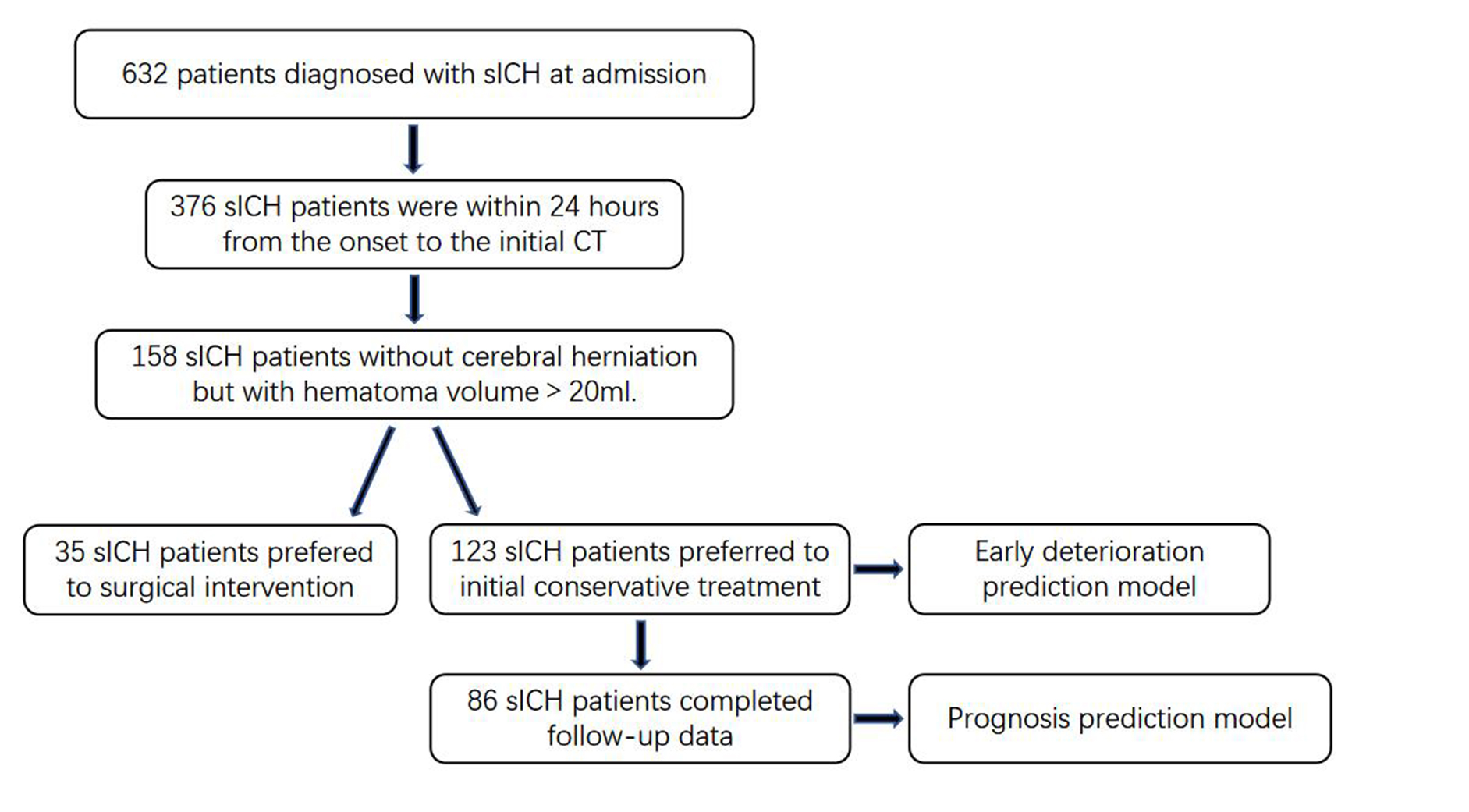

Supplement: Supplemental Figure 3 — ROC curve analysis between hematoma volume and poor prognosis in 1 year after stroke, AUC was.627,cutoff point was 24.8 ml (blue line). ROC curve analysis of prognosis prediction model, AUC was.792, the cutoff point was 4.5 (green line). [file Image_3.JPG]
